# High VEGFR1/2 expression levels are predictors of poor survival in patients with cervical cancer

**DOI:** 10.1097/MD.0000000000005772

**Published:** 2017-01-10

**Authors:** Yun-Zhi Dang, Ying Zhang, Jian-Ping Li, Jing Hu, Wei-Wei Li, Pei Li, Li-Chun Wei, Mei Shi

**Affiliations:** Department of Radiation Oncology, Xijing Hospital, The Fourth Military Medical University, Xi’an, Shaanxi, China.

**Keywords:** cervical cancer, overall survival, progression-free survival, vascular endothelial growth factor receptor 1, vascular endothelial growth factor receptor 2

## Abstract

The aim of the study to evaluate the prognostic significance of vascular endothelial growth factor receptor 1 and 2 (VEGFR1/2) expression levels and to correlate these levels with clinicopathological parameters in patients with cervical cancer.

Forty-two patients with International Federation of Gynecology and Obstetrics Stage IIB–IVB cervical cancer were analyzed between January 2011 and December 2012. RNA expression levels of VEGFR1/2 were assessed by branched DNA-liquidchip technology and immunohistochemistry. Associations between RNA expression levels, important clinicopathological parameters, and patient survival were statistically evaluated.

Higher VEGFR1/2 expression levels were predictive of poor overall survival (*P* = 0.009 and *P* = 0.024, respectively). Patients with higher VEGFR1 expression levels were associated with poorer progression-free survival than those with lower VEGFR1 expression levels (*P* = 0.043). In addition, patients with higher VEGFR1 expression levels were more likely to develop distant metastases than those with lower VEGFR1 expression levels (*P* = 0.049). Higher VEGFR2 expression levels were associated with larger tumor size (*P* = 0.037).

VEGFR1/2 expression levels were prognostic factors for patients with cervical cancer. Higher VEGFR1/2 expression levels were also predictive of poor overall survival.

## Abstract 2

Supplemental Digital Content is available in the text

## 1 Introduction

Cervical cancer is the second most common cancer and the third leading cause of cancer-related deaths among women worldwide.^[1]^ Although concomitant chemoradiotherapy has been recommended as the standard treatment for locally advanced cervical carcinoma since 1999, the 5-year survival rate of these patients is still around 70.0%.^[2]^ Therefore, new therapeutic targets are urgently needed.

Angiogenesis is a key pathological process in the development of malignant disease that potentially causes cancer progression and metastasis.^[3,4]^ The vascular endothelial growth factor (VEGF) family plays an important role in new vessel formation. Angiogenesis is mediated through VEGF binding to members of the VEGF receptor (VEGFR) family, which includes VEGFR1 (Flt-1), VEGFR2 (KDR/Flk-1), and VEGFR3 (Flt-4) in mammals. Among these receptors, VEGFR2 is considered the most important receptor for angiogenesis, as it binds all VEGFA isoforms, VEGFC, and VEGFD. Activated VEGFR2 induces simultaneous activation of the PLC-γ-Raf kinase-MEK-MAP kinase and PI3K-AKT pathways to induce cellular proliferation and endothelial cell survival.^[5,6]^ Shen et al^[7]^ also showed that VEGFR2 was associated with the vasculogenic capacity of precancerous stem cells. VEGFR2 overexpression has been reported in various types of cancer, including cancers of the head and neck, lungs, colon, uterus, ovaries, and breast.^[8,9]^ Among esophageal adenocarcinoma and squamous cell carcinoma specimens, 100.0% of the assessed tumors were VEGFR2-positive.^[10]^

However, no consistent conclusion has been drawn concerning the relationship between VEGFR2 expression and survival. In patients with breast cancer, VEGFR2 expression did not correlate with tumor histological grade, International Federation of Gynecology and Obstetrics (FIGO) stage, or survival.^[11]^ In contrast, patients with bladder cancer who expressed lower levels of VEGFR2 were associated with poorer recurrence-free survival than those who expressed higher levels of VEGFR2.^[12]^ Aucejo et al^[13]^ reported that upregulated VEGFR2 expression was associated with poor differentiation and tumor progression in patients with hepatocellular carcinoma. In patients with cervical cancer, Jach et al^[14]^ identified an almost linear relationship between the intensity of VEGFR2 expression and cervical intraepithelial neoplasia grading. Kuemmel et al^[15]^ demonstrated that soluble VEGFR2 expression in patient plasma increased significantly with disease progression (*P* = 0.014). VEGFR may function in malignant transformation and tumor growth in cervical cancer. However, the predictive value of VEGFR expression for the prognosis of patients with cervical cancer remains unclear.

In many types of cancer, high VEGFR1 expression is a predictor of poor survival. A meta-analysis^[16]^ showed that high VEGFR1 expression was associated with poor survival in patients with nonsmall cell lung cancer and improved survival was observed for patients with VEGFR1-negative esophageal cancer. In patients with breast cancer, high VEGFR1 expression levels were associated with an increased risk of death.^[17]^ To the best of our knowledge, only 1 cervical cancer study^[18]^ found that higher VEGFR1 expression levels were significantly associated with poorer progression-free survival (PFS) and overall survival (OS), using a cut-off level of 100 pg/mL. However, in this study, all patients had FIGO Stage IB1–IIB disease and the prognostic significance of VEGFR1 expression levels in locally advanced cervical cancer is still unclear.

We aimed to evaluate the prognostic significance of VEGFR1/2 expression in patients with cervical cancer. Moreover, we investigate the relationship between VEGFR1/2 expression and important clinicopathological parameters.

## 2 Materials and methods

### 2.1 Study population

Forty-two patients with FIGO Stage IIB–IVB cervical carcinoma^[18]^ were analyzed between January 2011 and December 2012. Histopathological diagnoses were established from biopsy specimens. The study was implemented with the approval of the local Institutional Review Boards. Patients with FIGO Stage IIB–IIIB disease were treated with external beam radiotherapy to the pelvis and intracavitary brachytherapy (n = 33) or surgery (n = 4). Patients with FIGO Stage IVB disease were treated with external beam radiotherapy to the pelvis, as well as the para-aortic and positive lymph nodes, and intracavitary brachytherapy. All patients were treated with 3-dimensional conformal radiotherapy or intensity-modulated radiotherapy using a 6 MV photon beam. For all patients, intravenous cisplatin was administered (40 mg/m^2^ weekly) concurrently during external beam radiotherapy treatments.

### 2.2 RNA expression levels of VEGFR1/2 detected by branched DNA-liquidchip technology

Fresh tissue specimens were collected by biopsy before treatment and stored in liquid nitrogen until required for this study. Detection of RNA expression was performed by Surexam Bio-Tech Co., Ltd. (Guangzhou, China). Fresh tissue specimens were processed according to the following steps. First, the sample was transferred to a fresh microcentrifuge tube. Second, 40 μL of the sample homogenate was incubated in a 96-well plate with buffer containing RNase-free water (18.5 μL), lysis solution (33.3 μL), blocking reagent (2.0 μL), capture beads (1.0 μL), and target gene-specific probes (5.0 μL), in each well, for 18 hours at 54°C on a shaker set at 750 rpm. Third, the hybridization mixture was transferred to a 96-well filter plate and washed 3 times with 250 μL of wash buffer (0.1× standard saline citrate and 0.03% lithium lauryl sulfate) to remove unbound RNA and other debris. Fourth, the samples used to detect bound target messenger RNA were incubated in 100 μL of pre-amplifier solution for 1 hour at 50°C, washed twice with 200 μL of wash buffer, incubated in 100 μL of amplifier solution for 1 hour at 50°C, washed twice with 200 μL of wash buffer, incubated in 100 μL of the labeled probe for 1 hour at 50°C, and washed twice with 200 μL of wash buffer. Finally, the samples were exposed with 100 μL of streptavidin-R-phycoerythrin conjugate solution for 30 minutes at 50°C. The fluorescence of each sample was analyzed using the Luminex 200 System (Luminex Corp., Shanghai, China). Beta-2-microglobulin, TATA box-binding protein, and transferrin receptor were used as control genes. Target gene messenger RNA expression levels were detected by Surexam Bio-Tech Co., Ltd. (Guangzhou, China) and compared to those in a database of Chinese cancer patients (Surexam Bio-Tech Co., Ltd., Guangzhou, China).

### 2.3 Immunohistochemistry of VEGFR1/2

Tissue sections (4 μm thick) were cut and incubated in a 3.0% hydrogen peroxide solution following citrate buffer (pH 6) to promote antigen retrieval. After the sections were incubated for 10 minutes at room temperature, primary antibodies against VEGFR1 (dilution: 1:200; Abgent Biotech Co., Ltd., Suzhou, China) and VEGFR2 (dilution: 1:200; CST Biological Reagents Co., Ltd., Shanghai, China) were added and incubated at 4^o^C overnight. Negative controls were run by omitting the primary antibodies.

Immunohistochemical stains were evaluated by 2 independent pathologists. VEGFR1/2 expression levels were evaluated based on the staining intensity and percentage of positive cells within the whole tissue section.^[19]^

### 2.4 Statistical analyses

VEGFR1/2 expression levels were scored into 3 categories: <25.0%, 25.0–75.0%, and ≥75.0%. As dichotomization of the <25.0% and ≥25.0% groups resulted in larger differences, this cut-off was used. The chi-square test was used to evaluate associations between biomarkers and other clinicopathological parameters. PFS was defined as the time from completion of radiotherapy to the date of disease progression. Similarly, OS was defined as the time from completion of radiotherapy to the date of death from any cause. PFS and OS were calculated using the Kaplan–Meier method. All statistical analyses were conducted using Statistical Package for the Social Sciences for Windows, software version 16.0 (SPSS Inc., Chicago, IL). A 2-tailed *P* < 0.05 was considered statistically significant.

## 3 Results

### 3.1 Patient and tumor characteristics

Forty-two patients with primary cervical cancer were included in this study. The median age of the patients was 47 (range, 29–71) years. Tumors from 41 patients (97.6%) had squamous histology, whereas adenocarcinoma was detected in 1 patient (2.4%). Twenty-one patients (50.0%) presented with lymph node metastasis at the time of diagnosis. Thirty-one patients (73.8%) were diagnosed with FIGO Stage IIIB cervical cancer. Patient and tumor characteristics are presented in detail in Table 1.

**Table 1:**
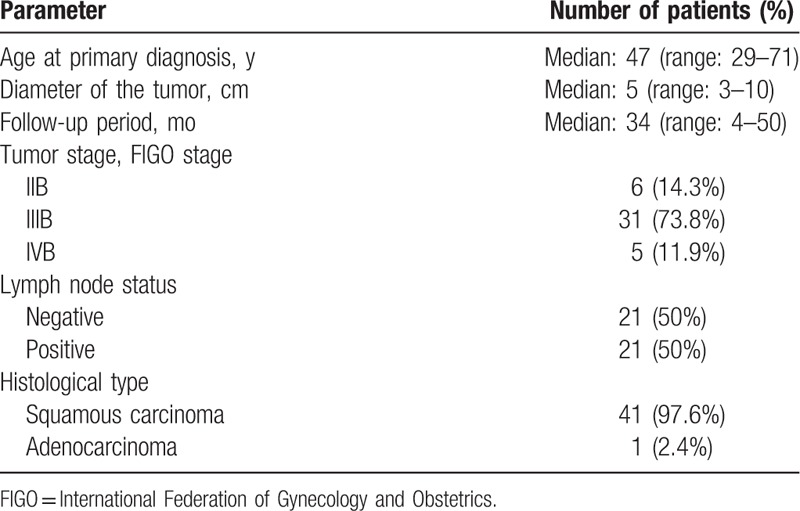
Patient characteristics.

### 3.2 Associations between VEGFR1/2 expression levels and clinical prognostic factors

Patients with poorly differentiated squamous carcinoma had higher VEGFR1 expression levels than patients with well or moderately differentiated disease (*P* = 0.031). Higher VEGFR2 expression levels were associated with a significantly larger tumor size (*P* = 0.037). Associations between VEGFR1/2 expression levels and age at primary diagnosis, FIGO stage, smaller lymph node diameters, the number of positive lymph nodes, gross tumor type, and primary response at the end of treatment were not statistically significant. The data are represented in detail in Table 2.

**Table 2:**
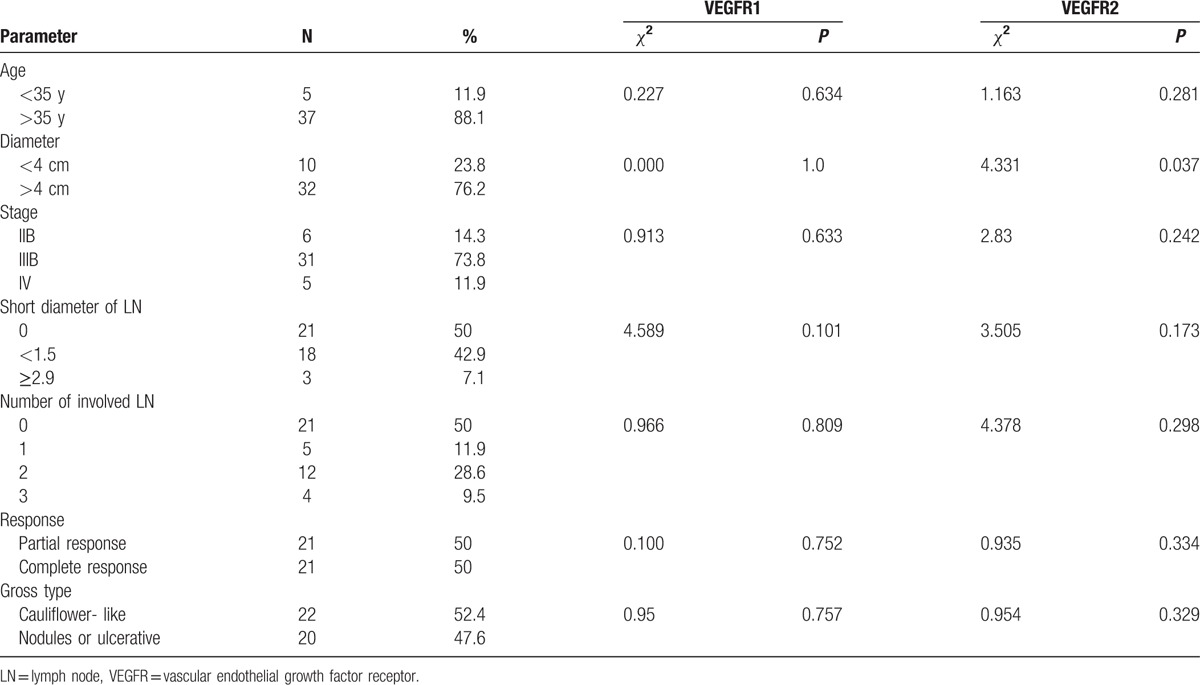
VEGFR1 and VEGFR2 expression levels and clinicopathological features.

### 3.3 Associations between VEGFR1/2 expression levels and patient survival

Patients were followed-up until April 2016, resulting in a median follow-up duration of 45 (range, 4–61) months. During this period, 18 patients (40.9%) experienced disease recurrence and 14 patients (31.8%) died. The estimated 3-year PFS and OS rates were 57.1% (95% confidence interval [CI]: 49.5–64.7%) and 71.3% (95% CI: 64.3–78.3%), respectively.

**Figure 1:**
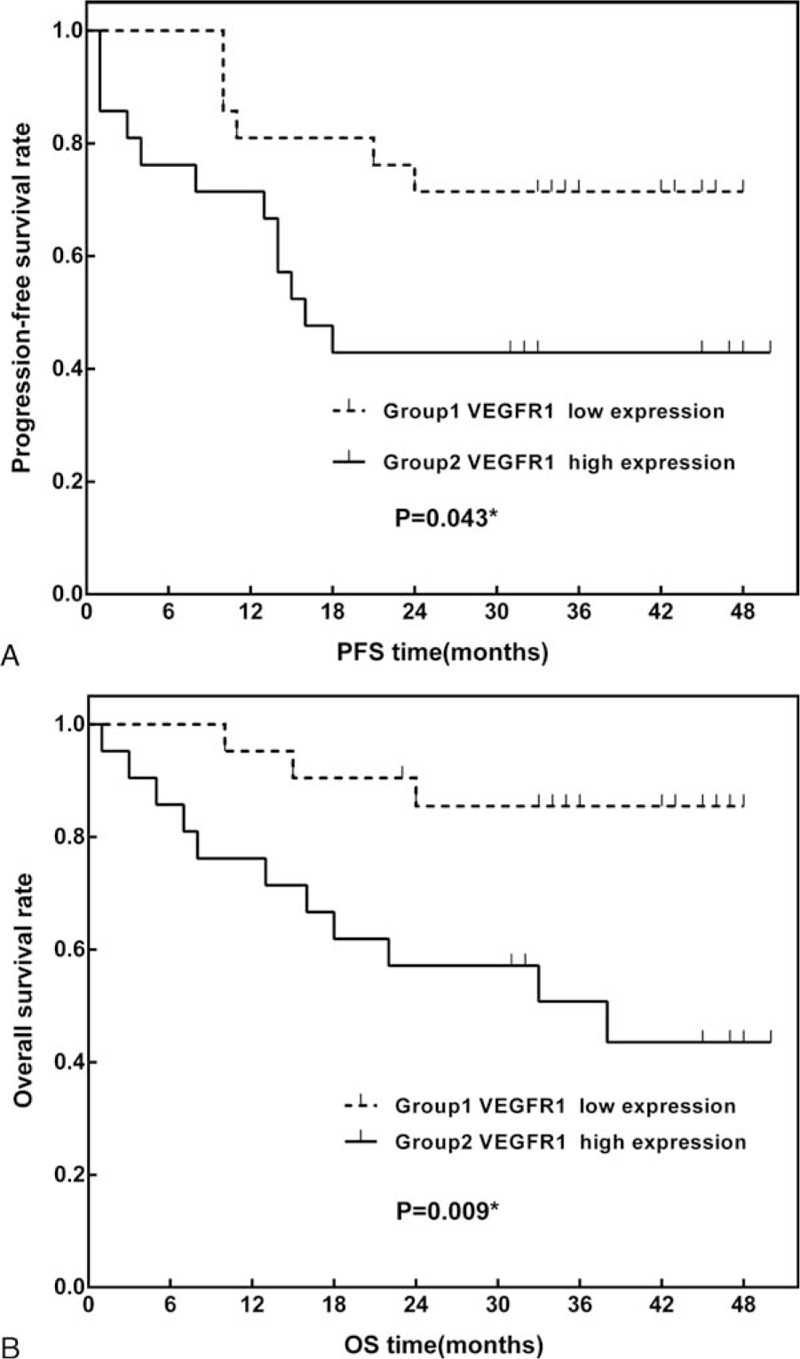
Kaplan–Meier curves of (A) 3-year progression-free survival (PFS) and (B) overall survival (OS) in patients stratified according to high and low vascular endothelial growth factor receptor 1 expression. OS = overall survival, PFS = progression-free survival.

Patients with higher VEGFR1 expression levels were associated with poorer PFS compared to those with lower VEGFR1 expression levels (*P* = 0.043). The estimated 3-year PFS rates were 85.4% (95% CI: 77.6–93.2%) and 57.1% (95% CI: 46.3–67.9%) for patients with low and high VEGFR1 expression levels, respectively (Fig. 1A). Higher VEGFR1 expression levels were also predictive of poor OS (*P* = 0.009). The estimated 3-year OS rates were 85.4% (95% CI: 77.6–93.2%) and 51.9% (95% CI: 40.9–62.9%) for patients with low and high VEGFR1 expression levels, respectively (Fig. 1B).

**Figure 2:**
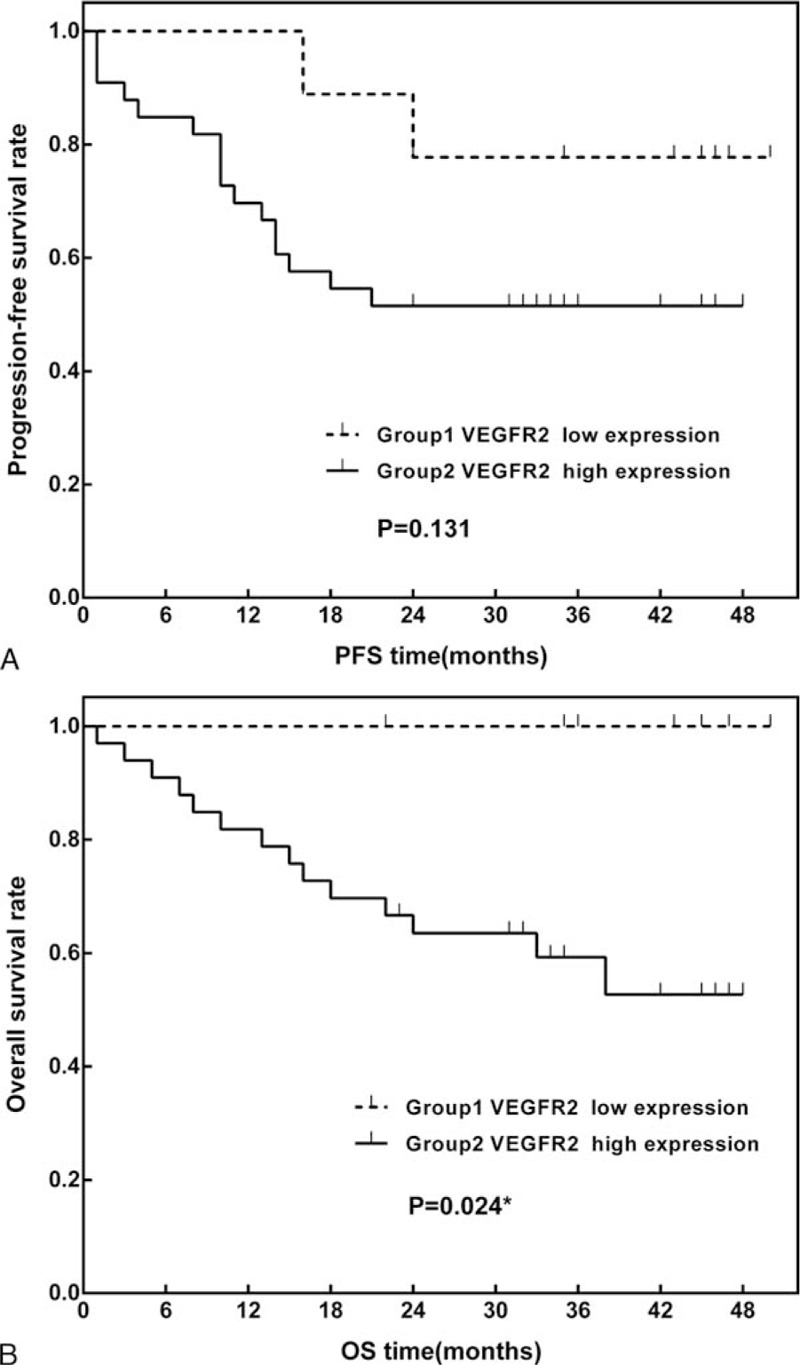
Kaplan–Meier curves of (A) 3-year progression-free survival (PFS) and (B) overall survival (OS) in patients stratified according to high and low vascular endothelial growth factor 2 expression. OS = overall survival, PFS = progression-free survival.

In the univariate analysis, VEGFR2 expression levels were not associated with PFS (*P* = 0.131; Fig. 2A). However, higher VEGFR2 expression levels were predictive of poor OS (*P* = 0.029). The estimated 3-year OS rates were 100.0% and 60.3% (95% CI: 51.7–68.9%) for patients with low and high VEGFR2 expression levels, respectively (Fig. 2B).

**Figure 3:**
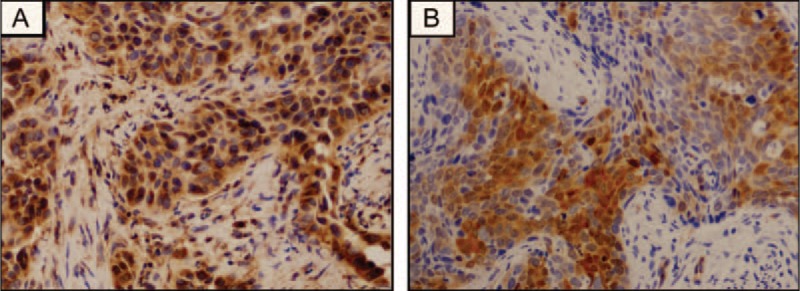
Immunostaining for VEGFR1 and VEGFR2 in cervical cancer. VEGFR1 expression was found in the cytoplasm of cervical cancer (A; × 200) lesions. VEGFR2 expression was found in the cytoplasm of cervical cancer (B; × 200) lesions.

By immunohistochemistry, there were 21 patients (50.0%) with moderate or high immunohistochemical expression of VEGFR1 (Fig. 3A). Patients with higher VEGFR1 expression levels were associated with poorer PFS and OS compared to those with lower VEGFR1 expression levels (*P* = 0.035 and *P* = 0.048, respectively). The moderate or high immunohistochemical expression rate of VEGFR2 was 81.0% (n = 34; Fig. 3B). Higher VEGFR2 expression levels were predictive of poor OS (*P* = 0.038). However, VEGFR2 expression levels were not associated with PFS (*P* = 0.068).

### 3.4 Associations between VEGFR1/2 expression levels and disease recurrence

Eighteen patients (40.9%) developed disease recurrence. Of these, 9 patients (50.0%) experienced distant metastasis, 8 patients (44.4%) experienced local recurrence, and 1 patient (5.6%) experienced both local recurrence and distant metastasis. Patients with higher VEGFR1 expression levels were more likely to experience distant metastasis compared to those with lower VEGFR1 expression levels (*P* = 0.046). Conversely, there was no relationship between disease recurrence and VEGFR2 expression levels (*P* = 0.182).

## 4 Discussion

Discoveries of altered molecular events in tumor cells are vital for revealing new and promising targets for treatments and improvements in cervical cancer outcomes. In the present study, the expressions of the angiogenic receptors VEGFR1 and VEGFR2 were investigated in cervical cancer tissue, along with their prognostic significance in patients with cervical cancer.

In our study, we found that patients with cervical cancer who expressed higher levels of VEGFR1 were associated with poorer PFS and OS. Patients with higher VEGFR1 expression levels were also more likely to develop distant metastasis (*P* = 0.049). In the past, VEGFR1 was less frequently investigated because it was not considered to be a signaling receptor or mediator of traditional VEGF-associated functions in endothelial cells.^[20]^ In 2005, Wey et al^[21]^ demonstrated that VEGFR1 could activate the extracellular regulated protein kinases (ERK1/2) signaling pathway to promote tumor cell migration and invasion in pancreatic carcinoma cell lines. Given our clinical data, we hypothesize that VEGFR1 expression levels may be associated with a high risk of tumor recurrence and poor survival. Further in vivo and in vitro molecular studies are needed to elucidate possible associations between VEGFR1 overexpression and treatment outcomes.

In the present study, higher VEGFR2 expression levels were predictive of poor OS. To the best of our knowledge, this is the first report to describe a negative prognostic value for higher VEGFR2 expression levels in patients with cervical cancer. A study by Adhemar et al^[9]^ reported that VEGFR2 overexpression was not associated with OS or local disease recurrence in patients with cervical adenosquamous carcinoma, but the VEGFR2 expression was associated with the absence of metastasis. Another report^[22]^ found that patients with cervical cancer who expressed higher levels of VEGFR2 were associated with better clinical responses (*P* = 0.02) using a 0.54 cut-off value from receiver operating characteristic curve analysis. The findings of the present study suggest that VEGFR2 is a new promising therapeutic target for patients with cervical cancer. Recently, biological and preclinical data showed that VEGFR2 inhibition could inhibit tumor-induced angiogenesis.^[23]^ Moreover, several VEGFR2 inhibitors are being evaluated in phase I–III clinical trials. Apatinib (YN968D1), a novel and potent VEGFR2 inhibitor, has displayed promising results for the treatment of patients with advanced or metastatic adenocarcinoma of stomach or gastroesophgeal in a phase III randomized trial.^[24]^

In this study, there was a significant association between higher VEGFR2 expression levels and a larger tumor size (*P* = 0.037). This may be explained by the fact that high VEGFR2 expression levels can promote tumor vascularization and cause cellular proliferation, although the possible mechanisms for this remain unclear. We were unable to correlate VEGFR1/2 expression levels with age at primary diagnosis, FIGO stage, gross tumor type, or primary response at the end of treatment. These data were in accordance with several previously published reports.^[18,25]^

There has been no consensus on the relationship between VEGFR1/2 expression levels and lymph node characteristics. In the present study, we found no association between VEGFR1/2 expression levels and smaller lymph node diameters or the number of positive lymph nodes. However, Kuemmel et al^[15]^ reported higher VEGFR2 expression levels in patients with positive lymph nodes compared to those with negative lymph nodes. In this study, VEGFR2 expression levels were detected by enzyme-linked immunosorbent assay and there were only 19 cervical cancer patients with positive lymph nodes.

In addition, VEGFA and VEGFB are ligands for VEGFR1 and/or VEGFR2. In this study, we also evaluated the expression levels of VEGFA and VEGFB. The moderate or high expression rate for VEGFA was 35.7% (n = 15), with 19 patients (45.2%) exhibiting moderate or high immunohistochemical staining for VEGFB in the cytoplasm of cervical cancer cells (Supplementary Figure S1). However, there were no significant correlations between VEGFA/B expression levels and clinicopathological parameters. According to the Kaplan–Meier method, there were also no significant correlations between VEGFA/B expression levels and patient survival. The expression levels of VEGFA/B did not correlate with those of their receptors, VEGFR1/2.

VEGFR3 is expressed in the lymphatic endothelium and is a key mediator of lymphangiogenesis. In our study, the moderate or high immunohistochemical staining rate for VEGFR3 in the cytoplasm and nucleus of cervical cancer cells was 59.5% (n = 25). No significant correlations were observed between VEGFA/B expression levels and patient survival.

In conclusion, this study revealed that VEGFR1/2 expression levels are significant prognostic factors for patients with cervical cancer. VEGFR1 expression was associated with distant metastasis, PFS, and OS, whereas the VEGFR2 expression was associated with tumor size and OS. These findings are potentially valuable for individualized treatments in the future.

## Supplementary Material

Supplemental Digital Content
