# Supplementary material for: High VEGFR1/2 expression levels are predictors of poor survival in patients with cervical cancer
Source: Medicine (Baltimore). 2017 Jan 10;96(1):e5772. doi: 10.1097/MD.0000000000005772 (PMC5228683; doi:10.1097/MD.0000000000005772)

**Supplementary Figure Legends**

Supplementary Figure 1 Immunostaining for VEGFA, VEGFAB and VEGFR3 in cervical cancer. VEGFA expression was found in the neclear of cervical cancer (A;░×░200) lesions. VEGFB expression was found in the cytoplasm of cervical cancer (B;░×░200) lesions. VEGFR3 expression was found in both the cytoplasm and nuclear of cervical cancer (B;░×░200) lesion


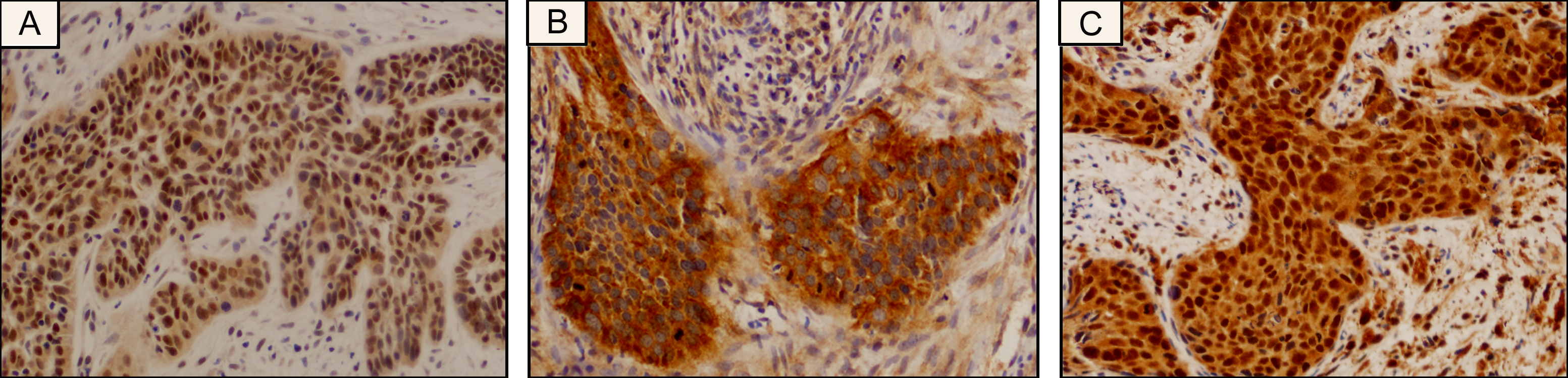

Supplement: Supplemental Digital Content [file medi-96-e5772-s001.doc]
